# Supplementary material for: Sister DNA Entrapment between Juxtaposed Smc Heads and Kleisin of the Cohesin Complex
Source: Mol Cell. 2019 Jul 25;75(2):224–237.e5. doi: 10.1016/j.molcel.2019.05.023 (PMC6675936; doi:10.1016/j.molcel.2019.05.023)
Supplement: Document S1. Figures S1–S6 [file mmc1.pdf]

Molecular Cell, Volume *75*

Supplemental Information

Sister DNA Entrapment between Juxtaposed Smc Heads and Kleisin of the Cohesin Complex

Christophe Chapard, Robert Jones, Till van Oepen, Johanna C. Scheinost, and Kim Nasmyth

Supplemental Figures:

Figure S1. Related to Figure 1 and 2

(A) Multiple sequence alignment indicating the lack of conservation of Smc residues which were mutated to cysteines.

(B) Cells containing indicated cysteine residues were grown in YPD at different temperatures (25°C, 30°C and 37°C). Coiled coils, E and J heads cysteine pairs do not rescue lethality of eco1(G211H) temperature sensitive mutant at 37°C.

(C) In vivo cysteine cross-linking of Smc1(N1192C)-HA6 and Smc3(R1222C)-PK6 proteins depends on both cysteine residues and BMOE. Crosslinking reaction was performed in vivo with BMOE. Cell protein extracts were separated by SDS-PAGE and analysed by western blot.

(D) In vivo cysteine cross-linking of Smc1(S161C)-HA6 and Smc3(K160C)-PK6 proteins depends on both cysteine residues and BMOE. Crosslinking reaction was performed in vivo with BMOE. Cell protein extracts were separated by SDS-PAGE and analysed by western blot.

(E) In vivo cysteine cross-linking of Smc1(K201C)-myc9 and Smc3(K198C)-PK6 proteins depends on both cysteine residues and BMOE. Crosslinking reaction was performed in vivo with BMOE. Cell protein extracts were separated by SDS-PAGE and analysed by western blot.

Figure S2. Related to Figure 3 and 4

(A) Smc1(K639C)-HA6 and Smc3(E570C, 968/969flagTEVx3)-PK6 or Smc1(K639C, N1192C)-HA6 and Smc3(E570C, 968/969flagTEVx3, R1222C)-PK6 proteins containing hinge and/or E heads cysteine pairs were cross-linked in vivo using BMOE. Complexes were immunoprecipitated on Smc3-PK6, cut by recombinant Tev protease, separated by SDS-PAGE and western blot. Note that on a western-blot, hinge-E-head double 4C cross-link is masked by hinge single 2C cross-link. Engineered TEV cleavage of Smc3 reveals the double crosslinking.

(B) Smc1 and Smc3-HaloTag proteins containing E heads, alternative J heads and/or coiled coils cysteine pairs were cross-linked in vivo with BMOE. Complexes were immunoprecipitated on Scc1-PK6, labelled with TMR ligand, separated by SDS-PAGE and quantified using in-gel fluorescence. Percentage of cross-link efficiency is given. Asterisk shows the location of the double crosslink.

(C) Smc1 and Smc3-HaloTag proteins containing E heads, J heads and/or coiled coils cysteine pairs were analyzed as in (B).

(D) Left panel: Smc1-HA, Smc3 and Scc1-PK proteins containing hinge and/or J heads cysteine pairs were crosslinked in vivo, immunoprecipitated on Scc1-PK6, separated by SDS-PAGE and analysed by western blot. Right panel: Smc1 and Smc3-HaloTag proteins containing hinge and/or J heads cysteine pairs were analysed as in (B). Percentage of the double cross-link efficiency is indicated in box.

(E-F) Coiled coils interactions in trimers. Smc1-HA, Smc3 and Scc1-pk6 proteins containing coiled coils cysteine pairs combined with cysteine pairs at the Smc3-Scc1 (C) or Smc1-Scc1 (D) interface

were cross-linked in vivo using BMOE. Complexes were immunoprecipitated on Scc1-PK6, separated by SDS-PAGE and semi-quantified by western blot. Percentage of cross-link efficiency is given.

Figure S3. Related to Figure 5

(A) FACS profiles for the experiment described in Figure 5A.

(B) Strains containing the Scc1 gene under the control of the WT Scc1 or Met3-repressible promoter as described in Figure 5B were grown in synthetic media lacking methionine or YPD.

(C) FACS profiles for the experiment described in Figure 5B.

(D) FACS profiles for the experiment described in Figure 5C.

(E) FACS profiles for the experiment described in Figure 5D.

(F) FACS profiles for the experiment described in Figure 5A.

(G) FACS profiles for the experiment described in Figure 5E.

(H) FACS profiles for the experiment described in Figure 5F.

(I) Strains containing Pds5-AID or Eco-AID do not grow on YPD + 5mM Auxin

Figure S4. Related to Figures 6 and 7

(A) Exemple of an independent repeat of the experiment shown in Figure 6A and showing CMs and CDs in exponentially growing strains containing cysteines in the hinge, E heads and/or Smc1/Scc1/Smc3 interfaces. Note that part of the blot shown in this panel is also shown in Figure 6C with a different contrast.

(B) Exemple of an independent repeat of the experiment shown in Figure 6B and showing CMs and CDs in exponentially growing strains containing cysteines in the hinge, J heads and/or Smc1/Scc1/Smc3 interfaces.

(C) Smc1-HA and Smc3 proteins containing hinge, E heads, J heads or coiled coils cysteine pairs were cross-linked in vivo using BMOE and analyzed as in Figure 7A.

(D) FACS profiles for the experiment described in Figure 7B.

(E) FACS profiles for the experiment described in Figure 7C.

Figure S5. Related to Figure 7

(A) Calibrated ChIP-seq profiles of Scc1-PK6 in the absence of Wpl1 and in the presence of WT Eco1 (blue) or the absence of both Wpl1 and functional Eco1 (orange). Sixteen yeast chromosomes profiles are centred on the CDEIII element.

(B) Calibrated ChIP-seq profiles of Scc1-PK6 on chromosomes I and IV in the absence of Wpl1, plus/minus functional Eco1.

Figure S6. Related to Figure 7

(A) Calibrated ChIP-seq profiles of Pds5-PK6 in the absence of Wpl1 and presence of WT Eco1 (blue) or the absence of both Wpl1 and functional Eco1 (orange). Sixteen yeast chromosomes profiles are centred on the CDEIII element.

(B) Calibrated ChIP-seq profiles of Pds5-PK6 on chromosomes I and IV in the absence of Wpl1, plus/minus functional Eco1.

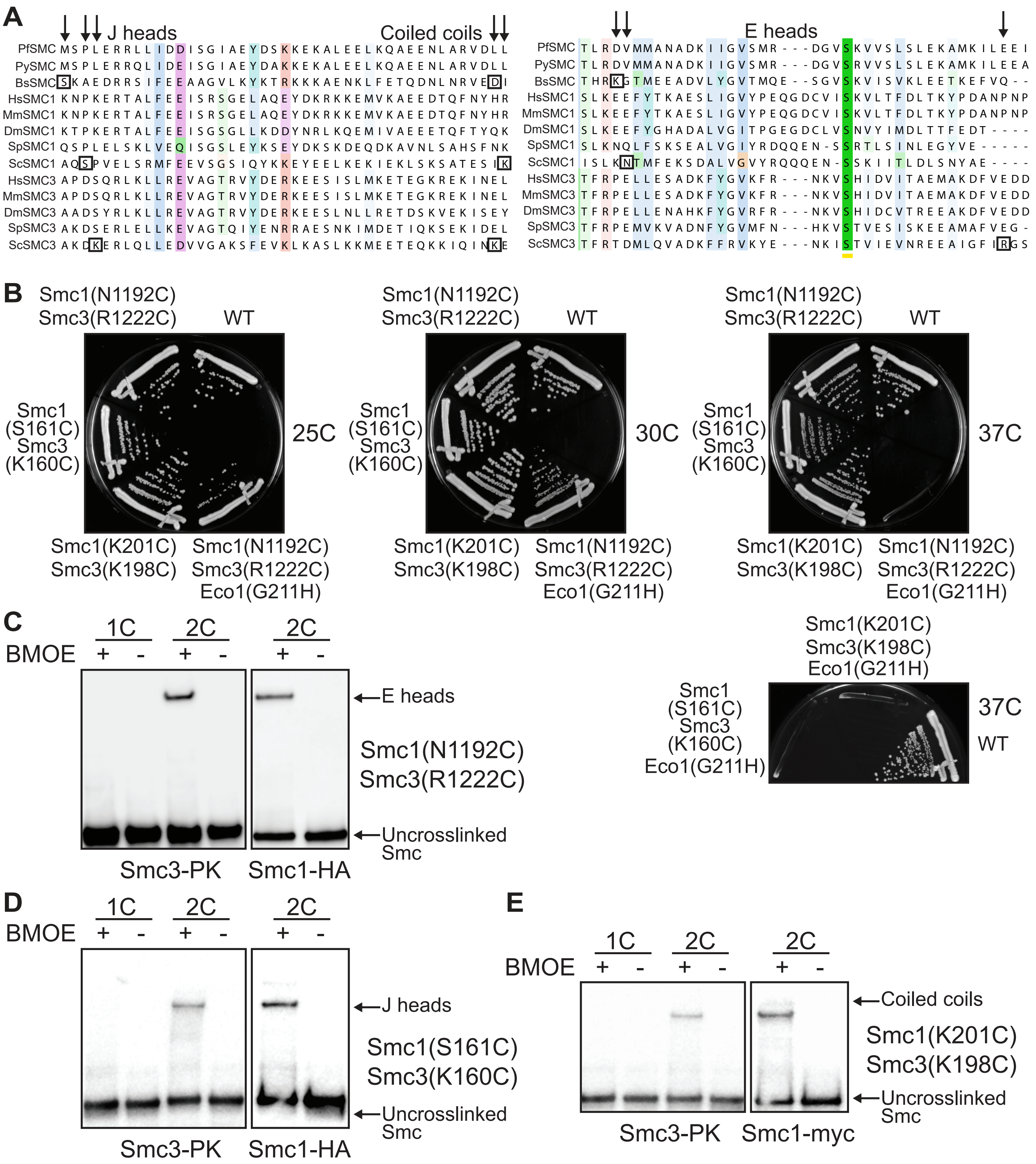
A
J heads
Coiled coils
E heads
PfSMC MSPLERRLLIDDISGIAEYDSKKEKALEELKQAEENLARVDLL
PySMC MSPLERRQLIDEISGIAEYDAKKEKALEELKQAEENLARVDLL
BsSMC SKAEDRRSIFEEAAGVLKYKTRKKKAENKLFETQDNLNRVEDI
HsSMC1 KNPKERTALFEEISRSGELAQEYDKRKKEMVKAEEDTQFNYHR
MmSMC1 KNPKERTALFEEISRSGELAQEYDKRKKEMVKAEEDTQFNYHR
DmSMC1 KTPKERTALFEEISGSGLLKDDYNRLKQEMIVAEEETQFTYQK
SpSMC1 QSPLELSKLVEQISGSLEYKSEYDKSKDEQDKAVNLSAHSFNK
ScSMC1 AQSPVELSRMFEEVSGSIQYKKEYEELKEKIEKLSKSATESIK
HsSMC3 APDSQRLKLLREVAGTRVYDERKEESISLMKETEGKREKINEL
MmSMC3 APDSQRLKLLREVAGTRVYDERKEESISLMKETEGKREKINEL
DmSMC3 AADSYRLKLLREVAGTRVYDERKEESLNLLRETDSKVEKISEY
SpSMC3 AKDSERLELLKEVAGTQIYENRRAESNKIMDETIQKSEKIDEL
ScSMC3 AKDKERLQLLEDVVGAKSFEVKLKASLKKMEETEQKKIQINKE
PfSMC TLRDVMMANADKIIGVSMR---DGVSKVVSLSLEKAMKILEEI
PySMC TLRDVMMANADKIIGVSMR---DGVSRVVSLSLEKAMKILEEA
BsSMC THRKGTMEEADVLYGVTMQE--SGVSKVISVKLEETKEFVQ--
HsSMC1 SLKEEFYTKAESLIGVYPEQGDCVISKVLTFDLTKYPDANPNP
MmSMC1 SLKEEFYTKAESLIGVYPEQGDCVISKVLTFDLTKYPDANPNP
DmSMC1 SLKEEFYGHADALVGITPGEGDCLVSNVYIMDLTTFEDT----
SpSMC1 SLKNQLFSKSEALVGIYRDQQEN-SSRTLSINLEGYVE-----
ScSMC1 ISLKNTMFEKSDALVGVYRQQQEN-SSKIITLDLSNYAE----
HsSMC3 TFRPELLESADKFYGVKFR---NKVSHIDVITAEMAKDFVEDD
MmSMC3 TFRPELLESADKFYGVKFR---NKVSHIDVITAEMAKDFVEDD
DmSMC3 TFRPELLENAHKFYGVRFR---NKVSHIDCVTREEAKDFVEDD
SpSMC3 TFRPEMVKVADNFYGVMFN---HKVSTVESISKEEAMAFVEG-
ScSMC3 TFRTDMLQVADKFFRVKYE---NKISTVIEVNREEAIGFIRGS
B
Smc1(N1192C) Smc3(R1222C)
WT
Smc1 (S161C) Smc3 (K160C)
25C
30C
37C
Smc1(K201C) Smc3(K198C)
Smc1(N1192C) Smc3(R1222C) Eco1(G211H)
Smc1(K201C) Smc3(K198C) Eco1(G211H)
Smc1 (S161C) Smc3 (K160C) Eco1(G211H)
C
1C
2C
BMOE
+
-
E heads
Smc1(N1192C) Smc3(R1222C)
Uncrosslinked Smc
Smc3-PK
Smc1-HA
D
J heads
Smc1(S161C) Smc3(K160C)
E
Coiled coils
Smc1(K201C) Smc3(K198C)
Smc3-PK
Smc1-myc

Sup. figure1

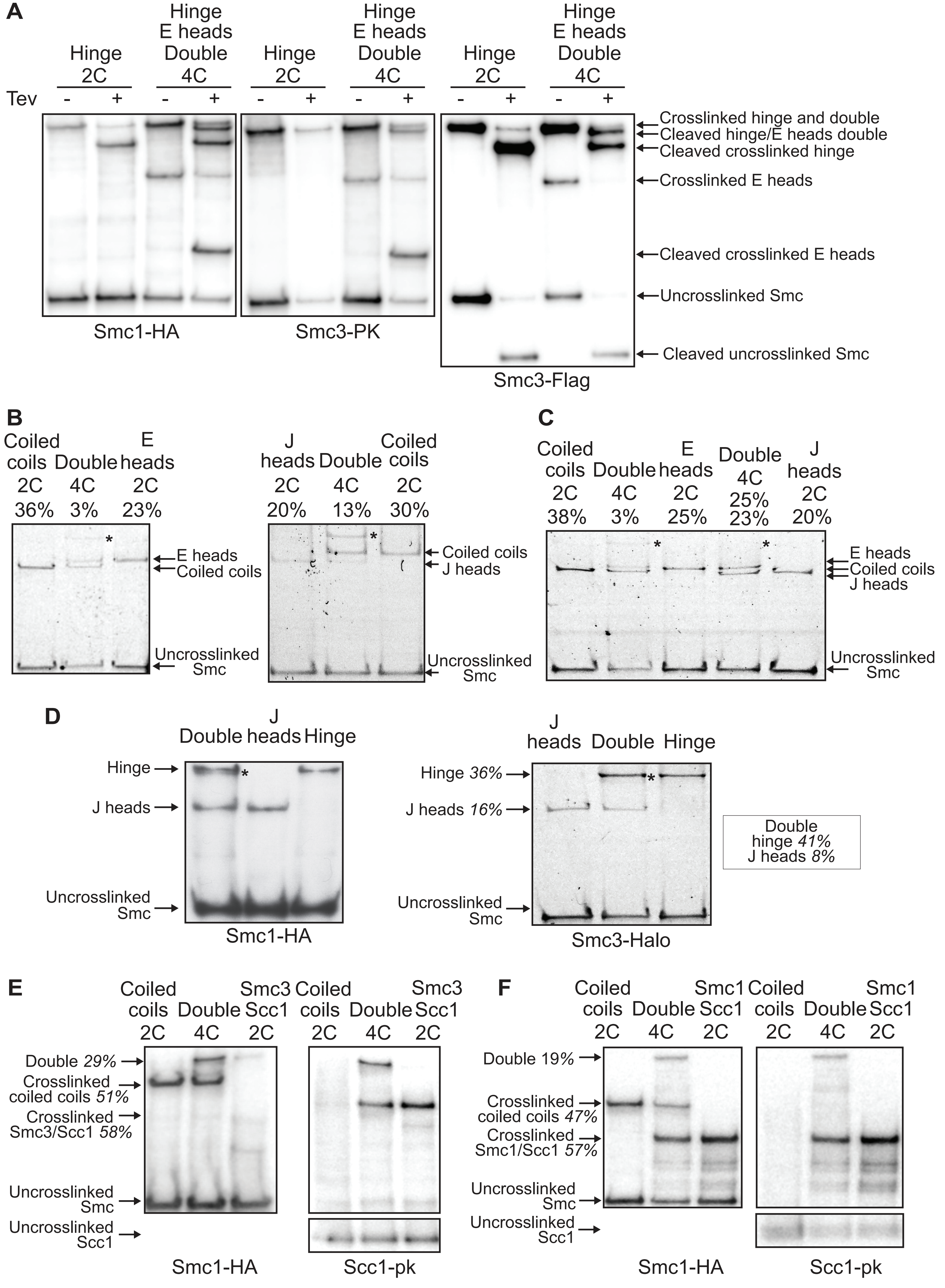
A
Hinge 2C
Hinge E heads Double 4C
Tev - + - +
Crosslinked hinge and double
Cleaved hinge/E heads double
Cleaved crosslinked hinge
Crosslinked E heads
Cleaved crosslinked E heads
Uncrosslinked Smc
Cleaved uncrosslinked Smc
Smc1-HA
Smc3-PK
Smc3-Flag
B
Coiled coils 2C 36%
Double 4C 3%
E heads 2C 23%
E heads
Coiled coils
Uncrosslinked Smc
J heads 2C 20%
Double 4C 13%
Coiled coils 2C 30%
Coiled coils
J heads
Uncrosslinked Smc
C
Coiled coils 2C 38%
Double 4C 3%
E heads 2C 25%
Double 4C 25% 23%
J heads 2C 20%
E heads
Coiled coils
J heads
Uncrosslinked Smc
D
Double
J heads
Hinge
Hinge
J heads
Uncrosslinked Smc
Smc1-HA
J heads
Double
Hinge
Hinge 36%
J heads 16%
Uncrosslinked Smc
Smc3-Halo
Double hinge 41% J heads 8%
E
Coiled coils 2C
Double 4C
Smc3 Scc1 2C
Double 29%
Crosslinked coiled coils 51%
Crosslinked Smc3/Scc1 58%
Uncrosslinked Smc
Uncrosslinked Scc1
Smc1-HA
Scc1-pk
F
Coiled coils 2C
Double 4C
Smc1 Scc1 2C
Double 19%
Crosslinked coiled coils 47%
Crosslinked Smc1/Scc1 57%
Uncrosslinked Smc
Uncrosslinked Scc1
Smc1-HA
Scc1-pk

Sup. figure2

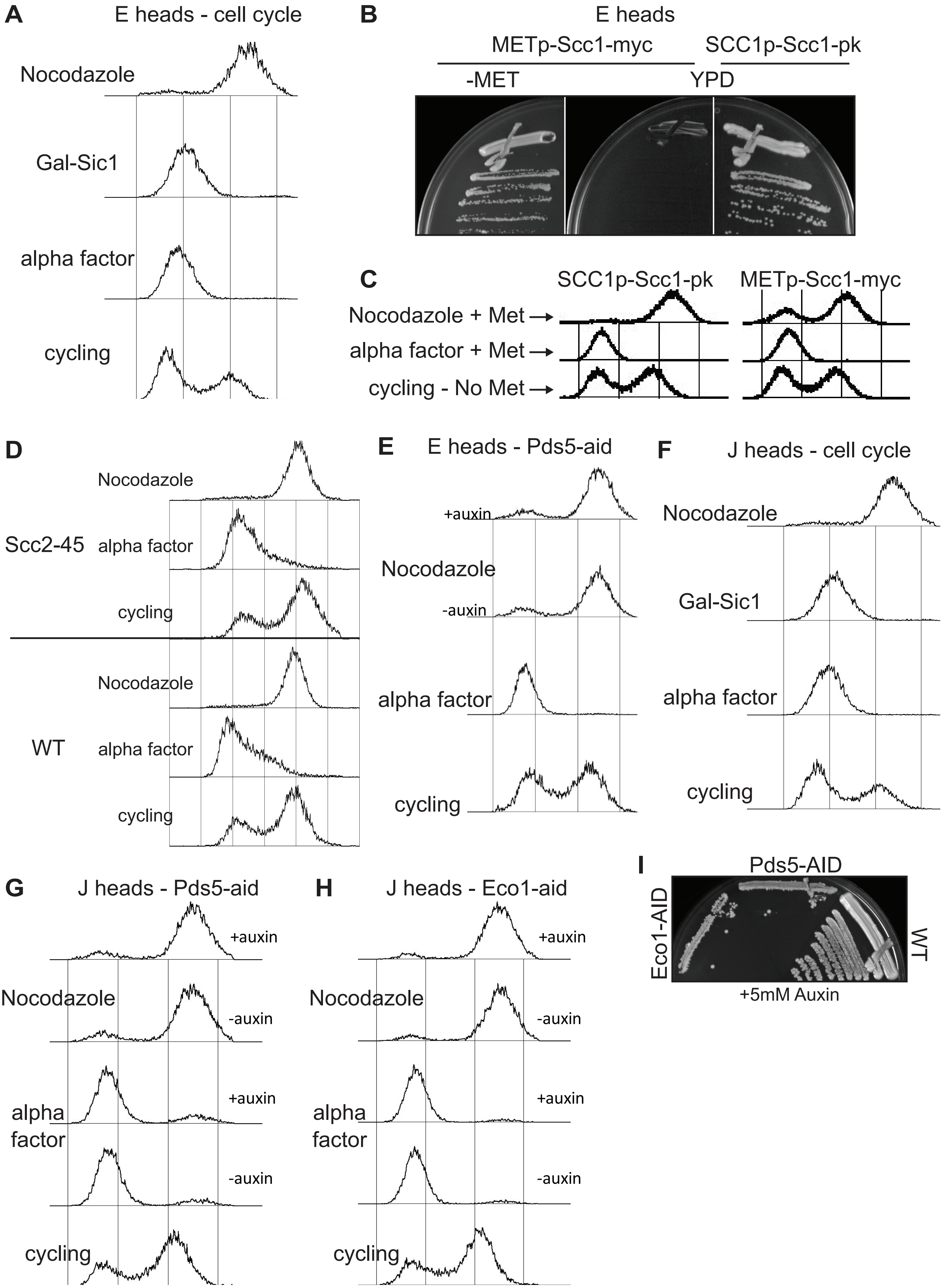
A
E heads - cell cycle
Nocodazole
Gal-Sic1
alpha factor
cycling
B
E heads
METp-Scc1-myc
SCC1p-Scc1-pk
-MET
YPD
C
SCC1p-Scc1-pk
METp-Scc1-myc
Nocodazole + Met
alpha factor + Met
cycling - No Met
D
Nocodazole
Scc2-45
alpha factor
cycling
Nocodazole
WT
alpha factor
cycling
E
E heads - Pds5-aid
+auxin
Nocodazole
-auxin
alpha factor
cycling
F
J heads - cell cycle
Nocodazole
Gal-Sic1
alpha factor
cycling
G
J heads - Pds5-aid
+auxin
Nocodazole
-auxin
+auxin
alpha factor
-auxin
cycling
H
J heads - Eco1-aid
+auxin
Nocodazole
-auxin
+auxin
alpha factor
-auxin
cycling
I
Pds5-AID
Eco1-AID
WT
+5mM Auxin

Sup. figure3

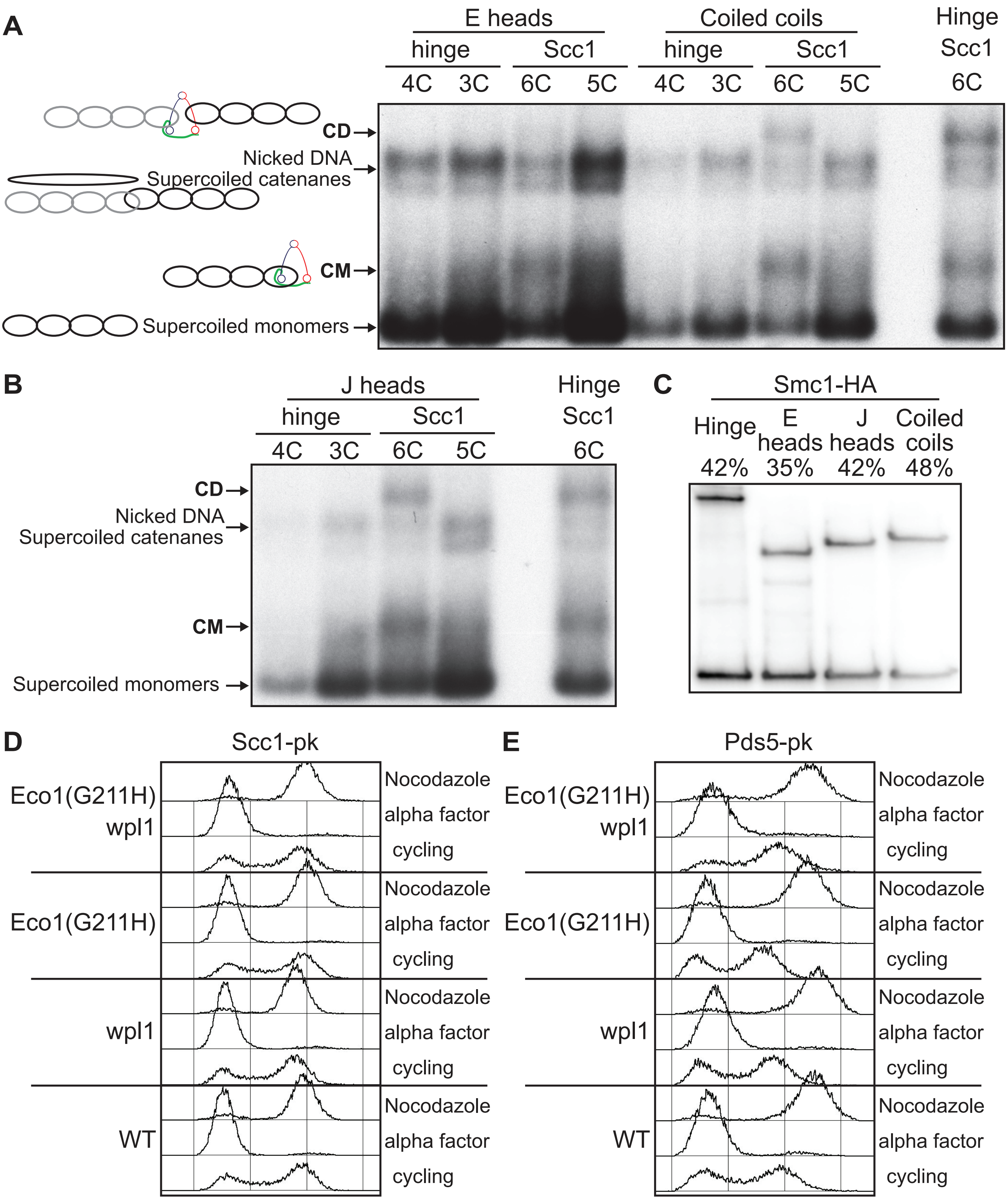

A
E heads
Coiled coils
hinge
Scc1
hinge
Scc1
Hinge
Scc1
4C 3C 6C 5C 4C 3C 6C 5C 6C
CD
Nicked DNA
Supercoiled catenanes
CM
Supercoiled monomers
B
J heads
hinge
Scc1
Hinge
Scc1
4C 3C 6C 5C 6C
CD
Nicked DNA
Supercoiled catenanes
CM
Supercoiled monomers
C
Smc1-HA
Hinge 42%
E heads 35%
J heads 42%
Coiled coils 48%
D
Scc1-pk
E
Pds5-pk
Eco1(G211H) wpl1
Eco1(G211H)
wpl1
WT
Nocodazole
alpha factor
cycling

Sup. figure 4

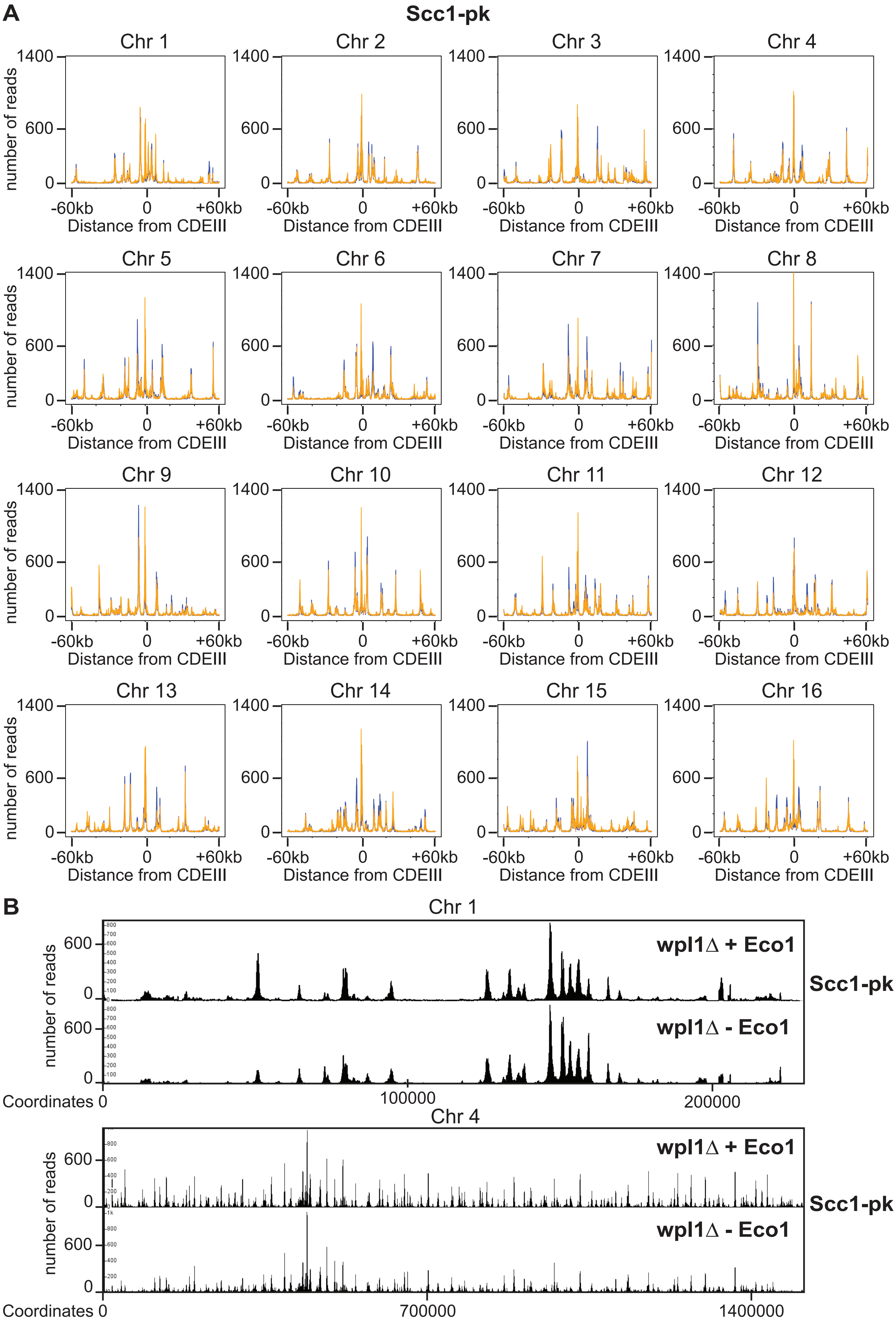
A
Scc1-pk
Chr 1
Chr 2
Chr 3
Chr 4
Chr 5
Chr 6
Chr 7
Chr 8
Chr 9
Chr 10
Chr 11
Chr 12
Chr 13
Chr 14
Chr 15
Chr 16
number of reads
1400
600
0
-60kb
0
+60kb
Distance from CDEIII
B
Chr 1
wpl1Δ + Eco1
wpl1Δ - Eco1
Scc1-pk
number of reads
Coordinates 0
100000
200000
Chr 4
700000
1400000

Sup. figure 5

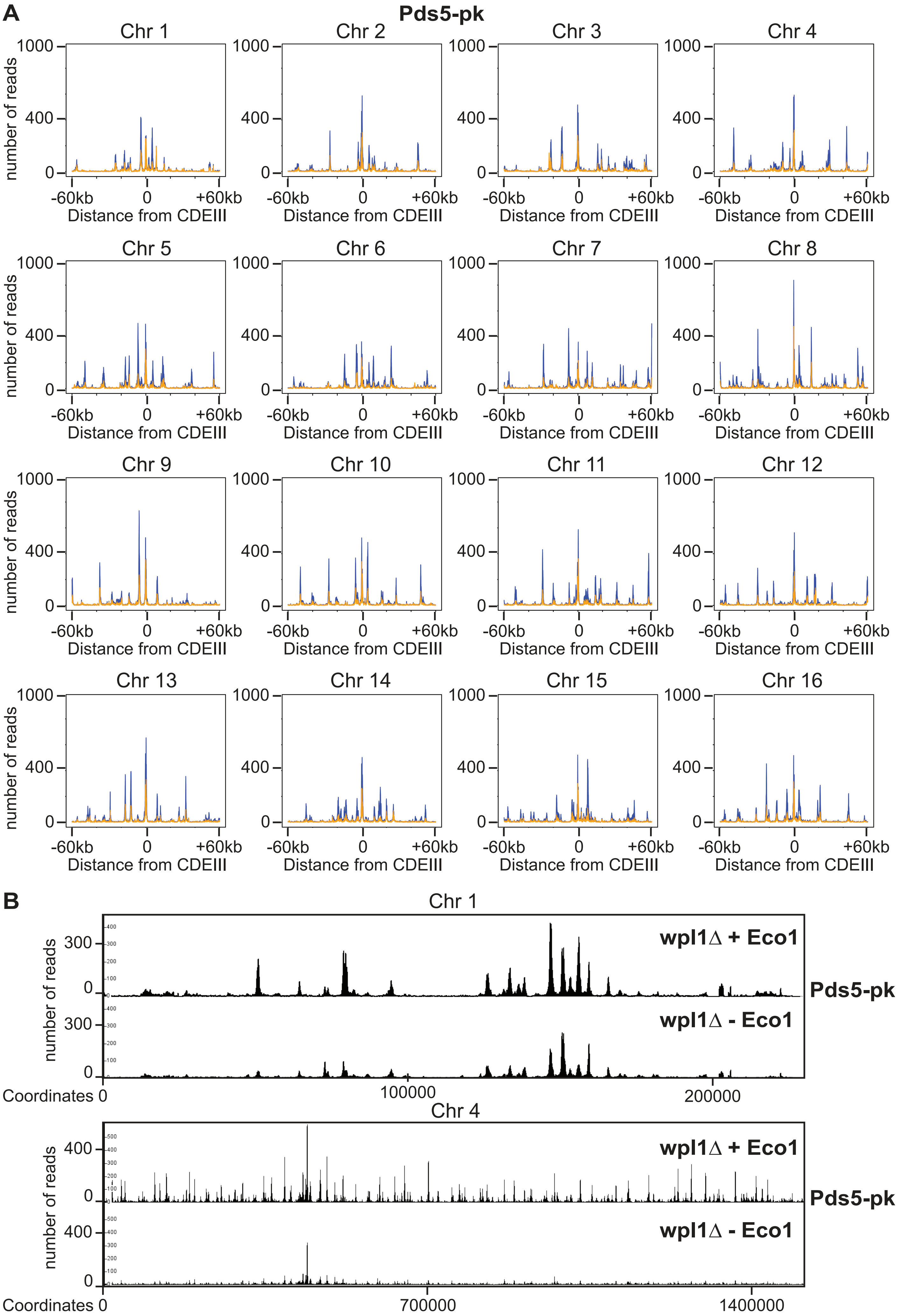
A
Pds5-pk
Chr 1
Chr 2
Chr 3
Chr 4
Chr 5
Chr 6
Chr 7
Chr 8
Chr 9
Chr 10
Chr 11
Chr 12
Chr 13
Chr 14
Chr 15
Chr 16
number of reads
1000
400
0
-60kb
0
+60kb
Distance from CDEIII
B
Chr 1
wpl1Δ + Eco1
wpl1Δ - Eco1
Pds5-pk
number of reads
300
0
Coordinates 0
100000
200000
Chr 4
400
700000
1400000

Sup. figure 6
